# Odor cueing during slow-wave sleep benefits memory independently of low cholinergic tone

**DOI:** 10.1007/s00213-017-4768-5

**Published:** 2017-11-08

**Authors:** Jens G. Klinzing, Sabine Kugler, Surjo R. Soekadar, Björn Rasch, Jan Born, Susanne Diekelmann

**Affiliations:** 1 Institute of Medical Psychology and Behavioral Neurobiology, University of Tübingen, Otfried Müller-Straße 25, 72076 Tübingen, Germany; 2 Graduate Training Centre of Neuroscience/IMPRS for Cognitive and Systems Neuroscience, University of Tübingen, 72074 Tübingen, Germany; 3 Applied Neurotechnology Laboratory, Department of Psychiatry and Psychotherapy, University Hospital of Tübingen, 72076 Tübingen, Germany; 4 Department of Psychology, University of Fribourg, 1700 Fribourg, Switzerland; 5 Centre for Integrative Neuroscience (CIN), University of Tübingen, 72076 Tübingen, Germany

**Keywords:** Memory consolidation, Sleep, Acetylcholine, Physostigmine, Odor reactivation

## Abstract

**Rationale:**

Sleep-dependent memory consolidation depends on the concerted reactivation of memories in the hippocampo-neocortical system. The communication of reactivated information from the hippocampus to the neocortex is assumed to be enabled by low levels of acetylcholine, particularly during slow-wave sleep (SWS). Recent studies suggest that the reactivation of memories does not only occur spontaneously but can also be externally triggered by re-presenting learning-associated cues during sleep.

**Objectives:**

Here we investigated whether the beneficial effect of cued memory reactivation during sleep depends on similar mechanisms as spontaneous reactivation, and specifically on low cholinergic tone.

**Methods:**

In two experimental nights, healthy volunteers learned a visuo-spatial memory task in the presence of an odor before going to sleep for 40 min. In one night, subjects were presented with the odor again during SWS, whereas in the other night they received an odorless vehicle. In half of the subjects, the availability of acetylcholine during sleep was increased by administering the acetylcholine-esterase inhibitor physostigmine.

**Results:**

Contrary to our hypothesis, increased cholinergic tone during sleep did not abolish the beneficial effect of odor cueing: memory performance was better after odor cueing compared to odorless vehicle, independent of physostigmine or placebo administration.

**Conclusions:**

This finding challenges the assumption that odor-cued and spontaneous memory reactivation rely on the same neuropharmacological mechanisms.

**Electronic supplementary material:**

The online version of this article (10.1007/s00213-017-4768-5) contains supplementary material, which is available to authorized users.

## Introduction

Successful long-term retention of memories depends on active consolidation mechanisms following initial encoding (McGaugh 2000; Dudai et al. 2015). The consolidation of new memories is assumed to be most effective during offline periods and particularly during sleep (Diekelmann and Born 2010; Stickgold and Walker 2013; Rasch and Born 2013). For declarative memories, a central component of consolidation is the repeated reactivation of learning-associated neuronal activity patterns originating in the hippocampus (Pavlides and Winson 1989; Wilson and McNaughton 1994; Nádasdy et al. 1999). Such reactivation is mainly observed during non-rapid eye movement (REM) sleep and occurs in temporal coordination with neocortical areas (Ji and Wilson 2007; Euston et al. 2007). This coordinated reactivation likely supports the extraction of regularities in recent experiences, their integration into existing memories, and the strengthening of neocortical long-term associations (Lewis and Durrant 2011; Landmann et al. 2014; Dudai et al. 2015).

While during the encoding of new experiences in the wake-state information flows mainly from neocortical areas to the hippocampal formation for rapid short-term storage, offline consolidation during sleep depends on a reversal of the direction of information flow from hippocampal areas back to neocortical sites (Buzsáki 1996). This shift in processing mode of the hippocampo-neocortical system from encoding information during wakefulness to consolidating memories during sleep is assumed to be regulated by the neurotransmitter acetylcholine (ACh) (Atherton, Dupret, and Mellor 2015; Hasselmo 1999). Cholinergic activity is high during wakefulness and lowest during slow-wave sleep (SWS) (Kametani and Kawamura 1990; Marrosu et al. 1995). Enhancing cholinergic signaling during sleep in humans by administering the acetylcholine-esterase inhibitor physostigmine has been shown to abolish the sleep-associated consolidation of declarative material (Gais and Born 2004). Blocking cholinergic signaling during wakefulness, in turn, improved declarative memory consolidation and impaired the encoding of new material (Rasch et al. 2006). Corroborating this evidence, increasing cholinergic activity by optogenetically stimulating septal neurons in mice diminished the occurrence of hippocampal sharp-wave ripples, which are considered a marker of sleep-dependent reactivation/consolidation; concurrently, this stimulation boosted theta oscillations, which are typically associated with wake encoding (Vandecasteele et al. 2014).

Reactivation of memory traces, which underlies the offline consolidation of declarative materials, does not only occur spontaneously during sleep but can also be triggered by external cues such as odors and sounds (Oudiette and Paller 2013). Stimulation with learning-associated cues, mainly during SWS, has been shown to enhance declarative as well as procedural and emotional types of memory (Rasch et al. 2007; Rudoy et al. 2009; Antony et al. 2012; Cairney et al. 2014; Schönauer et al. 2014; Cousins et al. 2014). For instance, presenting odor cues during SWS that have previously been associated with the learning of a visuo-spatial memory task enhances subsequent memory recall (Rasch et al. 2007), stabilizes memories against interfering information (Diekelmann et al. 2011), and accelerates spontaneous consolidation (Diekelmann et al. 2012). However, the neurophysiological mechanisms mediating cued memory reactivation are largely unknown. It could be hypothesized that the learning-associated stimulus facilitates the reactivation of cued memories via similar mechanisms as spontaneous reactivation. In support of this idea, cued memory reactivation during SWS biases hippocampal replay in favor of the memories associated with the cue in rats (Bendor and Wilson 2012). Furthermore, fMRI studies in humans suggest that, similar to spontaneous reactivations (Peigneux et al. 2004), cued reactivations go along with activation in para-/hippocampal areas (Rasch et al. 2007; van Dongen et al. 2012), in some cases together with neocortical areas (Diekelmann et al. 2011; Cousins et al. 2016). Connectivity studies showed an increase in functional connectivity between para-/hippocampal and neocortical areas in response to auditory cueing for declarative memory (van Dongen et al. 2012) and during later recall for procedural memory (Cousins et al. 2016). Evidence for a causal role of the hippocampus for declarative memory cueing comes from a study reporting that patients with bilateral hippocampal lesions failed to benefit from auditory cueing during sleep (Fuentemilla et al. 2013), although the same procedure enhanced memory performance in healthy subjects and patients with unilateral hippocampal lesions. Despite growing evidence from electrophysiological, imaging, and lesion studies, pharmacological studies on the role of specific neurotransmitters in cued memory reactivation are entirely lacking. Specifically, it is unclear whether neurotransmitters that are involved in spontaneous memory reactivation during sleep—and here mainly acetylcholine—are likewise implicated in cued memory reactivation. If cued memory reactivation during SWS relies on similar mechanisms as spontaneous reactivation, i.e., hippocampal replay and hippocampo-to-neocortical information flow, then the cueing effect might likewise depend crucially on low cholinergic tone during SWS.

In the present study, we tested this question by increasing cholinergic tone via administration of the acetylcholine-esterase inhibitor physostigmine during odor-cued reactivation of visuo-spatial memories in SWS. We hypothesized that the elevated levels of acetylcholine during SWS abolish the odor-cueing benefit.

## Methods

### Participants

A total of 29 men (mean age ± SD, 23.86 ± 2.47 years; range, 20–30) successfully completed the experiments. An additional eight subjects finished the experiment but were excluded from the analysis because they did not meet the criteria regarding sleep duration (*n* = 7) or learning performance (*n* = 1) (see below). None of the participants reported ongoing medication, health problems, current medical interventions, or a history of psychiatric, neurological, or sleep disorders. All participants passed a pre-experimental psychological diagnostic interview and a medical screening. Participants did not work on night shifts and did not have exam periods or other learning- or stress-intense occupations for at least 3 weeks prior to the experiment. On experimental days, daytime naps, extensive physical exercise, and the intake of alcohol or caffeine were prohibited. All subjects spent an adaptation night in the sleep laboratory to become accustomed to the experimental conditions. The study was approved by the local ethics committee of the medical faculty of the University Tübingen. All subjects gave written informed consent and were paid for participation.

### Experimental procedure

**Fig. 1:**
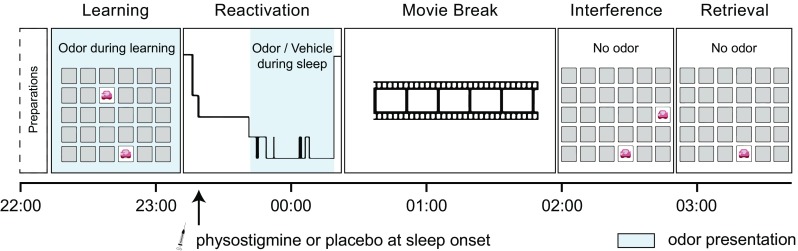
Experimental design. In the evening, all subjects learned a 2D object-location task under the presence of an odor. Starting at subsequent sleep onset, subjects received either an acetylcholine-esterase inhibitor (physostigmine group) or saline solution (placebo group) intravenously for 40 min. During the first 20 min of SWS, the learning-associated odor was presented again in one night (odor condition) and an odorless vehicle in the other night (vehicle condition). After a sleep period of ~ 40 min, subjects were woken up and watched a movie to allow for the effects of the drug to fade out. Subjects then learned an interference memory task and were finally tested on their recall of the original memory task

Subjects were randomly assigned to one of two groups and spent two nights in the laboratory. Subjects in the physostigmine group (*n* = 15) received an intravenous physostigmine infusion in both nights, whereas subjects in the placebo group (*n* = 14) received a placebo infusion in both nights. During one of the nights, all subjects were presented with learning-associated odor cues during SWS; during the other night, they were presented with an odorless vehicle, in counter-balanced order (Fig. 1). Each experimental night started at 20:30 h with the placement of polysomnographic electrodes and the intravenous catheter to the participant’s non-dominant arm. At 22:00 h, all subjects learned a 2D object-location task in the presence of the experimental odor. Starting at around 23:15 h, subjects slept for about 40 min (min. 30 min, max. 90 min), including about 20 min of SWS (min. 15 min, max. 30 min). If a participant did not fall asleep within 60 min or did not reach at least 15 min of SWS within 90 min of sleep, the experiment was discontinued. Administration of physostigmine dissolved in saline solution (physostigmine group) or pure saline solution (placebo group) was started at sleep onset (defined as the first epoch of S1 followed by S2). The experimental odor or vehicle was presented for the entire duration of SWS in alternating on/off blocks of 30 s to reduce habituation. Participants were woken up after the max. of 30 min of odor/vehicle stimulation during SWS or if substantial arousals occurred. To allow potential aftereffects of the drug to fade out, all subjects watched a movie for 1.5 h after awakening. Participants then learned an interference object-location task to test for memory stability and were finally tested for recall of the original object-location task.

Odor/vehicle stimulation and physostigmine/placebo administration during sleep were performed in a double-blinded fashion. The odor was delivered by a computer-controlled olfactometer via a nasal mask. The experimental odor was isobutyraldehyde (99%) diluted in 1,2-propanediol at a concentration of 1:50. The intravenous infusion contained 0.25 ml *Anticholium* (containing physostigmine salicylate) in 17 ml saline solution, which was delivered across 40 min (starting at sleep onset) at a rate of 25.5 ml per hour. Physostigmine is an acetylcholine-esterase inhibitor, suppressing the enzymatic breakdown of acetylcholine, with an elimination half-life of about 20–30 min (Aquilonius and Hartvig 1986; Hartvig et al. 1986).

### Memory task

In a 2D object-location task, subjects learned the locations of 15 card pairs, resembling the game “concentration” (Rasch et al. 2007; Diekelmann et al. 2011 2012). The card pairs depicted animals and everyday objects and were presented on a computer screen in a 5 × 6 matrix. During learning, the locations of all 15 card pairs were presented twice. For each card pair, the first card was presented for 1 s, then both cards for 3 s, followed by a 3-s inter-trial break. During the subsequent immediate recall test, the first card of each pair was presented and the subjects had to indicate the second card location with the computer mouse. Independent of the subject’s response, the correct location of both cards was then shown again for 2 s. The immediate recall procedure was repeated until the subjects reached a criterion of 60% correct responses. Participants who did not reach the criterion after a maximum of six runs were dismissed from the study. The experimental odor was presented time-locked to the presentation of the cards throughout the learning session. For the two experimental nights, two parallel versions of the task were used showing different pictures at different locations.

Before the recall session, subjects learned an interference task to test for memory stability. The learning of the interference task was identical to learning of the original task, with the same 15 card pairs, but the second card of each pair being located at a different position (similar to an A–B, A–C interference paradigm, with A, B, and C referring to locations; Fig.1). Moreover, interference learning included only one immediate recall run to ensure comparable interference input for all subjects. About 30 min after interference learning, final recall of the original memory task was tested in only one recall run. The first card of each pair was presented and subjects had to indicate the location of the second card. No odor cues were presented during the interference task or final recall. Memory performance was calculated as the percentage of correctly recalled card locations at final recall relative to the number of correct card locations during the last immediate recall run at learning (i.e., criterion run).

### Sleep recordings, physiological parameters, and control variables

During sleep, standard polysomnographic recordings were obtained, including electroencephalography (EEG, from positions F3, F4, C3, C4, P3, and P4) referenced to the averaged mastoids (M1, M2), electromyography, and electrooculography. An electrocardiogram was recorded to monitor the participants’ heart rate during drug application. Sleep recordings were scored online and offline according to standard criteria (Rechtschaffen and Kales 1968) as sleep stages S1–S4 (with stages S3 and S4 comprising SWS), REM sleep, and wakefulness.

Blood pressure and heart rate were assessed at five time points during each experimental night (before learning, before sleep, after waking, after the interference task, and after final recall). Blood samples were taken in order to determine cortisol concentration at three time points (before sleep, after waking, and between interference task and final recall) to assess individual stress levels. To control for general alertness, a vigilance test and a set of questionnaires were administered at three time points (before learning, after the interference task, and after final recall). In the vigilance task, subjects had to respond as quickly as possible to a red dot appearing every 2–10 s for a total of 10 min on the left or right side of a computer screen and reaction times (in ms) were assessed. Subjects rated their subjective sleepiness on the Stanford Sleepiness Scale (Hoddes et al. 1973), their general mood on the short version of the Multidimensional Mood State Questionnaire (Steyer et al. 1994), and the presence or absence of 27 potential side effects of physostigmine (e.g., sweating, blurred vision, headache). Before and after learning, an odor detection test was performed to ensure olfactory sensitivity in all participants.

### EEG power spectral analysis and detection of spindles and slow oscillations

EEG signals were recorded at a sampling rate of 200 Hz and bandpass-filtered between 0.16–35 Hz. The power spectrum was estimated over subsequent 5-s segments (Hanning window with an overlap of 0.9 times the window length) for sleep stages S2 and SWS (i.e., combined sleep stages S3 and S4). Spindles and slow oscillations were detected in sleep stages S2–S4, using the open source toolbox SpiSOP (www.spisop.org; see Online Resource 1 for a more detailed description) and adopting the detection criteria from Mölle and colleagues (Mölle et al. 2011). Slow- and fast-spindle frequencies were defined individually for each dataset by peaks in the power spectrum of the averaged EEG channels F3/F4 between 9 and 12 Hz for slow spindles and of channels C3/C4 between 12 and 15 Hz for fast spindles. Slow oscillations were detected in the EEG recorded from F3/F4 after bandpass-filtering the signal between 0.3 and 3.5 Hz. For sleep spindles, the parameters density, duration, and maximal envelope were analyzed. For slow oscillations, duration, amplitude, up-to-down slope, and down-to-up slope were analyzed. In order to assess potential changes in these parameters due to the odor stimulation, we compared 10-s time windows before odor onset (“odor off”) and after odor onset (“odor on”).

### Statistical analysis

Memory performance, physiological parameters, sleep data, and control variables were analyzed using analyses of variance (ANOVAs) with the between-subject factor “physostigmine/placebo” and the within-subject factor “odor/vehicle.” For the power analysis, we introduced the additional within-subject factor “S2/SWS.” For the analysis of spindle and slow-oscillation events, we introduced the additional within-subject factor “odor off/odor on.” For all electrophysiological data, we followed an interquartile range outlier rejection rule (lower threshold: Q1–2.2 × (Q3–Q1); upper threshold: Q3 + 2.2 × (Q3–Q1)) (Hoaglin and Iglewicz 1987). Effect sizes are provided as partial eta squared (*η*_*p*_^2^) or Cohen’s *d*. Control parameters were Bonferroni-corrected for the number of tested measures. A *p* value of ≤ 0.05 was considered significant.

## Results

**Fig. 2:**
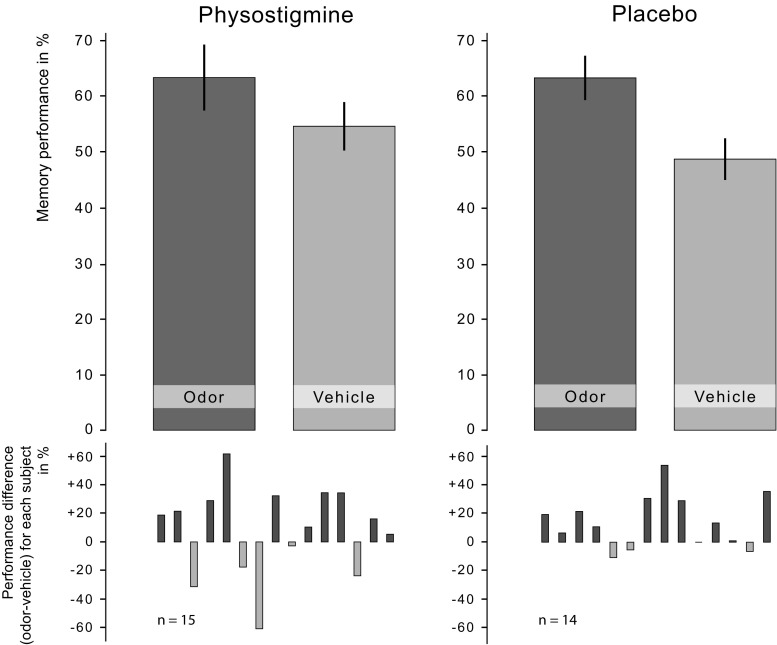
Memory performance after odor cueing under physostigmine. Odor cueing during SWS improved memory performance as compared to vehicle, independent of physostigmine administration (“odor/vehicle” main effect: *p* = 0.026). Top bars show the means ± s.e.m. of memory performance at final recall relative to the learning performance (with learning set to 100%) in the odor and vehicle conditions of the physostigmine and placebo groups, respectively. Small bottom bars show each participant’s performance difference between the odor and vehicle conditions, with positive values corresponding to better performance in the odor condition and negative values corresponding to better performance in the vehicle condition

Odor cueing during SWS improved memory performance in both the physostigmine and placebo group. Subjects remembered significantly more card pairs in the odor condition compared to the vehicle condition independently of the experimental group (main effect “odor/vehicle”: *p* = 0.026, *η*_*p*_^2^ = 0.17; mean ± s.e.m., placebo: odor 63.65 ± 3.84% vs. vehicle 48.97 ± 3.73%; physostigmine: odor 63.71 ± 6.05% vs. vehicle 54.91 ± 4.27%; Fig. 2). Contrary to our hypothesis, this benefit of odor cueing was not affected by the administration of physostigmine (interaction “physostigmine/placebo” × “odor/vehicle”: *p* = 0.56). However, post hoc tests comparing memory performance in the odor and vehicle conditions in the different groups showed that the odor effect was more consistent in the placebo group (odor vs. vehicle: *p* = 0.013, *d* = 0.77) and failed to reach significance in the physostigmine group (odor vs. vehicle: *p* = 0.31, *d* = 0.27). Physostigmine per se did not change overall memory performance (main effect “physostigmine/placebo”: *p* = 0.48).

There were no significant differences in performance between groups or conditions during initial learning as well as during interference learning (all *p* > 0.079, Table 1). The administration of physostigmine resulted in slight changes of sleep patterns (Table 2). Subjects in the physostigmine group tended to spend less time (in percent of total sleep time) in S4 (11.09 ± 3.22 vs. 18.86 ± 3.34%, *p* = 0.105, *η*_*p*_^2^ = 0.09, for main effect “physostigmine/placebo”) and spent more time in S3 (39.76 ± 2.82 vs. 28.90 ± 2.92%, *p* = 0.012, *η*_*p*_^2^ = 0.21, for main effect “physostigmine/placebo”), corresponding to an overall reduction in sleep depth. There was no effect of physostigmine on sleep stages 1 and 2 or time awake after sleep onset (all *p* > 0.24). Odor presentation during sleep did not change any of the sleep patterns (main effect “odor/vehicle” and interaction “odor/vehicle” × “physostigmine/placebo”: all *p* > 0.38). Total sleep time did not differ between groups or conditions, neither in the experimental night (all *p* > 0.36) nor in the night before the experiment (self-reported, all *p* > 0.17).

**Table 1:** Performance in the visuo-spatial memory task

	Physostigmine		Placebo	
	Odor	Vehicle	Odor	Vehicle
Learning performance	10.40 ± 0.27	10.80 ± 0.46	9.64 ± 0.25	10.29 ± 0.35
Learning trials	2.47 ± 0.31	2.13 ± 0.35	2.57 ± 0.42	2.43 ± 0.42
Recall performance	6.67 ± 0.67	5.93 ± 0.50	6.14 ± 0.39	5.07 ± 0.45
Interference learning	8.53 ± 0.92	8.33 ± 0.81	7.93 ± 0.84	7.43 ± 0.82

Learning performance (absolute number of cards recalled in the last learning trial), learning trials (repetitions needed to reach learning criterion), recall performance (absolute number of cards correctly indicated at final recall), and interference learning performance (number of cards recalled during immediate recall of the interference task) for each experimental group and condition (means ± s.e.m.)

**Table 2:** Sleep parameters and odor stimulations

	Physostigmine		Placebo	
	Odor	Vehicle	Odor	Vehicle
TST (in min)	46.93 ± 2.95	46.17 ± 2.56	47.32 ± 3.35	43.68 ± 3.73
S1 (in %)	5.21 ± 1.15	5.57 ± 1.28	5.82 ± 0.90	5.46 ± 0.70
S2 (in %)	41.37 ± 3.15	41.21 ± 2.23	46.32 ± 2.66	44.20 ± 3.19
S3 (in %)*	39.71 ± 3.42	39.81 ± 3.69	27.36 ± 3.18	30.44 ± 2.93
S4 (in %)	10.79 ± 3.58	11.39 ± 2.95	19.60 ± 3.55	18.12 ± 4.51
Wake (in %)	2.75 ± 1.74	1.90 ± 1.77	0.86 ± 0.42	1.53 ± 1.06
Stimulations	21.27 ± 0.32	20.53 ± 0.73	22.36 ± 0.79	20.86 ± 0.73

*TST* total sleep time, *S1–S4* relative time spent in each sleep stage, *Wake* relative time spent awake after sleep onset, and number of odor stimulations (means ± s.e.m.). *p = 0.012 for ANOVA main effect “physostigmine/placebo.” No other significant effects of group or condition

Estimates of EEG power reflected the group differences in sleep architecture with a significant reduction of slow-oscillation power in the physostigmine group at all three electrode sites (main effect “physostigmine/placebo”: frontal *p* = 0.021, *η*_*p*_^2^ = 0.20; central: *p* = 0.019, *η*_*p*_^2^ = 0.21; parietal: *p* = 0.025, *η*_*p*_^2^ = 0.19) and a marginally significant reduction in delta band power (frontal: *p* = 0.065, *η*_*p*_^2^ = 0.14; central: *p* = 0.040, *η*_*p*_^2^ = 0.16; parietal: *p* = 0.058, *η*_*p*_^2^ = 0.14, see Online Resource 2). No other main effects or interactions reached significance, except for an expected main effect “S2/SWS” for practically all frequency bands and electrodes, and an interaction “S2/SWS” × “odor/vehicle” for frontal electrodes (*p* = 0.046, *η*_*p*_^2^ = 0.16), indicating higher slow-oscillation power during S2 in the odor condition compared to the vehicle condition (all other *p* > 0.06). Analysis of sleep spindle and slow-oscillation events did not reveal any effects of physostigmine administration (all *p* > 0.06).

In the physostigmine group, heart rate was slightly lower in the beginning of the experimental night (interaction “physostigmine/placebo” × “time”: *p* < 0.01, η_p_^2^ = 0.18). There were no differences between groups or conditions in blood pressure (all *p* > 0.57) and cortisol levels (all *p* > 0.16) as well as in vigilance (all *p* > 0.10), subjective sleepiness (all *p* > 0.37), mood (all *p* > 0.77), or any of the side-effect ratings (all *p* > 0.15). There was also no difference in the number of odor/vehicle stimulations during SWS in any of the groups and conditions (all *p* > 0.12). Accuracy rates in the odor detection test were very high (overall average of ~ 95%).

## Discussion

In the present study, we investigated whether low levels of acetylcholine during SWS are essential for the beneficial effects of cued memory reactivation, similar to spontaneous sleep-dependent reactivation. Contrary to our hypothesis, increasing the availability of acetylcholine during SWS by physostigmine did not abolish the improving effect of odor cueing on memory.

Acetylcholine has been proposed to enable the information flow from hippocampal to neocortical sites for long-term memory consolidation during sleep (Hasselmo 1999). A previous study from our group found a complete abolition of the beneficial effect of sleep on memory consolidation after increasing the availability of acetylcholine by the administration of physostigmine (Gais and Born 2004). Animal studies further suggest that increased acetylcholine activity during sleep reduces hippocampal replay in the form of spontaneous sharp wave-ripple events (Vandecasteele et al. 2014) and blocks the propagation of reactivated information to the neocortex (Hasselmo and Schnell 1994; Douchamps et al. 2013). Following the assumption that the beneficial effect of cued memory reactivation during sleep relies on similar mechanisms as spontaneous reactivation, i.e., on influencing the hippocampo-to-neocortical information flow, we hypothesized that increasing the cholinergic tone during sleep would eliminate the cueing effect. Our finding of a preserved odor-cueing effect under physostigmine administration suggests that cued memory reactivation might not act by targeting the information flow from hippocampus to neocortex. We propose two alternative pathways by which odor cueing might improve memory performance independent of hippocampo-to-neocortical communication.

The effect of cued memory reactivation might rely on an immediate strengthening of associations within the hippocampus, without necessarily affecting neocortical representations. The 2D object-location task applied in the present study requires relatively item-specific memory, with little room for generalization or abstraction. In this task, cueing may allow for memory benefits resulting solely from intra-hippocampal mechanisms. Indeed, olfactory processing areas are directly and strongly connected to hippocampal areas (Zelano and Sobel 2005), with learning-associated odor cues possibly triggering hippocampal replay and neuronal strengthening. Brain imaging studies in humans, using olfactory cueing of the same 2D object-location task, observed increases in hippocampal activation in response to the odor cue during SWS compared to wake (Rasch et al. 2007). Plastic changes in the hippocampal formation, particularly involving connections between dentate gyrus and CA3 as well as recurrent CA3-CA3 associative networks (Kumaran et al. 2016), may be sufficient to strengthen the respective memories. Although spontaneous hippocampal sharp wave-ripple events can be reduced by elevated acetylcholine levels (Vandecasteele et al. 2014), a reinforcing effect of acetylcholine has been observed on pyramidal cell excitability and neural plasticity in both dentate gyrus and CA hippocampal subregions (Prince et al. 2016). For example, in vitro recordings have shown that elevated cholinergic tone constitutes a necessary condition for long-term potentiation (LTP) in CA1 (e.g., Isaac, Buchanan, Muller, and Mellor 2009). Such increased intra-hippocampal plasticity may counteract the reduction of overall replay events. The remaining replay events may be biased toward the cued information when externally reactivated (Bendor and Wilson 2012), leading to an increase in the relative number of task-related hippocampal reactivation events. The task-related reactivated memories may then be strengthened through increased intra-hippocampal plastic processes, improving the overall performance level.

Alternatively, odor cueing might directly trigger memory reactivation in distributed neocortical memory networks, bypassing the hippocampus. Although olfactory input has privileged access to the hippocampal formation, olfactory information is also relayed to a multitude of other areas, such as thalamus and various neocortical regions (Royet and Plailly 2004; Zelano and Sobel 2005). Furthermore, olfactory information becomes integrated with multimodal sensory as well as higher-order input in the piriform cortex due to its unusually dense interconnectedness similar to other association areas (Johnson et al. 2000; Haberly 2001). Activity emanating from the piriform cortex as a relay hub may trigger coherent activity in other cortical areas and may thereby strengthen distributed memory representations associated with the odor. Interestingly, associative LTP in the piriform cortex has been shown to benefit from enhanced cholinergic signaling (Patil et al. 1998). A recent study has further demonstrated that parietal cortical networks quickly assume functional support of spatial learning tasks already after a few learning repetitions (Brodt et al. 2016). Consequently, the 2D object-location task of our study may sufficiently profit from neocortical sleep-associated consolidation for behavioral effects to become evident, without essential contribution of the hippocampus.

These two alternative pathways are not mutually exclusive. Instead of affecting the communication between hippocampus and neocortex, odor cueing may trigger strengthening of associations in these two structures in a simultaneous but otherwise independent manner. An fMRI study, employing a very similar learning and odor-cueing paradigm as the present study, found both hippocampal and neocortical activation increases in response to the odor cue during SWS (Diekelmann et al. 2011). This study could not, however, differentiate between neocortical involvement as a result of hippocampal reactivation and independent contributions of the two structures. Another fMRI study reported increased parahippocampo-neocortical connectivity during auditory cueing (van Dongen et al. 2012), yet, the employed connectivity analysis could not establish the direction of information flow and might be confounded by neural activity time-locked to the cue. Finally, Cousins et al. (2016) showed an increase in connectivity between the hippocampus and task-related neocortical regions during testing of a motor learning task following auditory cueing during sleep. Unfortunately, this study did not assess brain activation patterns during sleep, which may additionally diverge between procedural and declarative memory. Auditory cueing may further differ from olfactory cueing in the underlying neural mechanisms, considering the substantial anatomical differences between the olfactory and auditory systems.

The above considerations are highly speculative and future studies should systematically test the outlined models (purely hippocampal, purely neocortical, and independent hippocampal and neocortical contributions). For example, it would be promising to investigate whether memory retrieval recruits different neural structures after odor cueing under elevated acetylcholine levels than after cueing under normal low cholinergic conditions. Furthermore, sophisticated imaging techniques could directly assess differential activation patterns during odor cueing in SWS under high and low cholinergic tone.

It should be noted that despite an overall main effect of odor cueing, the odor effect in our study seemed to be mainly driven by the placebo group. However, while the difference between the odor and vehicle condition in the physostigmine group was descriptively smaller and not per se significant, this difference was still of moderate effect size (*d* = 0.27) and the absence of an interaction indicates that the odor effect did not differ substantially between both groups. In further support of a general odor-cueing effect, we observed an increase in slow-oscillation power during S2 in the odor condition, independent of physostigmine administration. This effect points toward a general increase in slow-oscillation power through odor reactivation, which may become evident only during lighter sleep stages with lower baseline slow-oscillation power. Although these findings should be interpreted with caution, we believe that the pattern of results tentatively suggests that the role of acetylcholine in odor-cued memory reactivation may at least not be large. In future studies, larger samples would be necessary to detect a potentially smaller effect of physostigmine on cued memory reactivation.

At a first glance, our findings appear to contradict the results by Gais and Born (2004) since they do not replicate the effect of physostigmine abolishing sleep-dependent consolidation in the vehicle conditions. However, we did in fact not expect an effect of spontaneous sleep-dependent consolidation after a sleep period as short as 40 min. We have previously shown that 40 min of sleep is not enough to yield a spontaneous behavioral sleep benefit in the 2D object-location task, with a benefit only emerging with additional memory cueing (Diekelmann et al. 2011, 2012). This does not mean that there is no spontaneous reactivation during these 40 min of sleep, yet the behavioral effect seems to be dependent on the additional cued reactivation. We deliberately chose the 40-min sleep interval here because we aimed to isolate the odor-cueing effect from the spontaneous sleep consolidation effect. Accordingly, we did not expect a differential effect of physostigmine in the vehicle conditions.

A limitation of the present study may result from the employed memory task. Whereas Gais and Born (2004) used a word-pair learning task, we applied a visuo-spatial memory task and introduced an interference version of the task before retrieval testing to probe memory stability. Since different experimental tasks might rely on different neuronal substrates, the potential of increased acetylcholine to influence reactivation/consolidation processes may differ between tasks. Furthermore, it is possible that a higher dose of physostigmine over longer time intervals might have yielded stronger effects. Although we applied an overall lower dose of physostigmine over a shorter time than Gais and Born (2004), we are confident that physostigmine was physiologically active in our study, as evidenced by the expected reduction in sleep depth, reflected in a shift toward lighter sleep stages in line with Gais and Born (2004) and a reduction in EEG power in the slow oscillation and delta frequency bands. Nevertheless, future studies should test higher doses and aim to establish a precise dose-response curve for the effects of physostigmine on memory cueing during sleep.

## Electronic supplementary material

ESM 1 (DOC 31 kb)

ESM 2 (DOC 74 kb)
