# Supplementary material for: Odor cueing during slow-wave sleep benefits memory independently of low cholinergic tone
Source: Psychopharmacology (Berl). 2017 Nov 8;235(1):291–9. doi: 10.1007/s00213-017-4768-5 (PMC5748395; doi:10.1007/s00213-017-4768-5)
Supplement: Supplementary file 1 — (DOC 31 kb) [file 213_2017_4768_MOESM1_ESM.doc]

**Electronic Supplementary Material 1**

**Odor cueing during slow wave sleep benefits memory independently of low cholinergic tone**

**Journal: Psychopharmacology**

Jens G. Klinzing, Sabine Kugler, Surjo R. Soekadar, Björn Rasch, Jan Born, Susanne Diekelmann

Corresponding author:

Jens G. Klinzing jens.klinzing@uni-tuebingen.de, Tel: +49 7071 29 88932

Institute of Medical Psychology and Behavioral Neurobiology, University of Tübingen

**Supplementary Materials and Methods: EEG power spectral analysis and detection of spindles and slow oscillations**

EEG data from sleep stages S2-S4 were analyzed after visual exclusion of epochs with muscle or eye artifacts. Data were recorded at a sampling rate of 200 Hz and were bandpass-filtered between 0.16-35 Hz. One dataset was highpass-filtered at 0.5 instead of 0.16 Hz to eliminate low-frequency artifacts. In one case, electrode C3 and F3 were excluded from power and slow oscillation analysis due to low-frequency artifacts. Power spectral density was estimated over the frequency bands 0.1-1 Hz (SO), 1-4 Hz (delta), 4-8 Hz (theta), 9-12 Hz (slow spindles), and 12-15 Hz (fast spindles). Estimates were calculated over 5-sec segments (Hanning window with an overlap of 0.9 times the window length), averaged across time within sleep stage S2 and SWS (i.e. combined sleep stages S3 and S4), averaged over contralateral electrodes, and subsequently log-transformed. Inter-individual power differences were normalized using each participant’s theta band power, for which there was no change to be expected.

Spindles and slow oscillations were detected in sleep stages S2-S4 using the open source toolbox SpiSOP (www.spisop.org) and adopting the detection criteria from Mölle and colleagues (Mölle et al., 2011). Slow and fast spindle center frequencies were defined individually for each dataset by computer-assisted visual determination of peaks in the power spectrum of the averaged EEG channels F3/F4 between 9 and 12 Hz for slow spindles and of channels C3/C4 between 12 and 15 Hz for fast spindles. The change in spindle peak frequencies between the two experimental nights were generally small (0.29 ± 0.06 Hz for slow spindles, 0.21 ± 0.04 Hz for fast spindles) and frequencies were averaged across both nights for each subject. This resulted in a mean frequency across subjects of 10.64 ± 0.14 Hz for slow spindles and 13.27 ± 0.09 Hz for fast spindles. Each dataset was bandpass-filtered around the individual center frequencies ± 1.5 Hz but with the passed bands never extending beyond 8-12 Hz for slow spindles and 12-16 Hz for fast spindles. A spindle was registered if the smoothed root-mean-square of the filtered signal (calculated with an 0.2 sec sliding window) exceeded an amplitude 1.5 standard deviations above the mean for a time window of 0.5-3 sec and exceeded an amplitude 1.75 standard deviations above the mean at least once.

Slow oscillations were detected in the EEG recorded from F3/F4 after bandpass-filtering the signal between 0.3 and 3.5 Hz. A slow oscillation was registered if i) its negative peak exceeded 1.25 times the subject's average negative peak amplitude, ii) it showed a positive-to-negative peak amplitude difference of at least 1.25 times the subject's average positive-to-negative peak amplitude difference, and iii) if its duration was between 0.8 and 2 s (corresponding to a frequency between 0.5 and 1.2 Hz).

All recordings were performed using BrainAmp amplifiers and BrainVision recording software (Brainproducts GmbH, Gilching, Germany).
