# Supplementary material for: Odor cueing during slow-wave sleep benefits memory independently of low cholinergic tone
Source: Psychopharmacology (Berl). 2017 Nov 8;235(1):291–9. doi: 10.1007/s00213-017-4768-5 (PMC5748395; doi:10.1007/s00213-017-4768-5)
Supplement: Supplementary file 2 — (DOC 74 kb) [file 213_2017_4768_MOESM2_ESM.doc]

**Electronic Supplementary Material 2**

**Odor cueing during slow wave sleep benefits memory independently of low cholinergic tone**

**Journal: Psychopharmacology**

Jens G. Klinzing, Sabine Kugler, Surjo R. Soekadar, Björn Rasch, Jan Born, Susanne Diekelmann

Corresponding author:

Jens G. Klinzing jens.klinzing@uni-tuebingen.de, Tel: +49 7071 29 88932

Institute of Medical Psychology and Behavioral Neurobiology, University of Tübingen

**Supplementary Table 1 EEG power estimates**

|  |  | **Physostigmine** | | | | **Placebo** | | | |
| --- | --- | --- | --- | --- | --- | --- | --- | --- | --- |
|  |  | Odor |  | Vehicle |  | Odor |  | Vehicle |  |
| **SO frontal** *† | S2 | 2.17 | ± 0.08 | 2.14 | ± 0.09 | 2.43 | ± 0.07 | 2.34 | ± 0.04 |
|  | SWS | 2.76 | ± 0.10 | 2.76 | ± 0.12 | 3.01 | ± 0.10 | 3.02 | ± 0.08 |
| **SO central** * | S2 | 2.01 | ± 0.07 | 1.95 | ± 0.07 | 2.20 | ± 0.06 | 2.15 | ± 0.03 |
|  | SWS | 2.67 | ± 0.10 | 2.65 | ± 0.11 | 2.88 | ± 0.09 | 2.90 | ± 0.08 |
| **SO parietal** * | S2 | 1.90 | ± 0.07 | 1.88 | ± 0.07 | 2.06 | ± 0.06 | 2.02 | ± 0.03 |
|  | SWS | 2.63 | ± 0.09 | 2.64 | ± 0.10 | 2.81 | ± 0.08 | 2.80 | ± 0.05 |
|  |  |  |  |  |  |  |  |  |  |
|  |  | **Physostigmine** | | | | **Placebo** | | | |
|  |  | Odor |  | Vehicle |  | Odor |  | Vehicle |  |
| **delta frontal** ^#^ | S2 | 1.67 | ± 0.05 | 1.66 | ± 0.06 | 1.81 | ± 0.04 | 1.75 | ± 0.03 |
|  | SWS | 2.18 | ± 0.07 | 2.21 | ± 0.09 | 2.34 | ± 0.08 | 2.35 | ± 0.07 |
| **delta central** * | S2 | 1.55 | ± 0.04 | 1.51 | ± 0.04 | 1.63 | ± 0.03 | 1.62 | ± 0.02 |
|  | SWS | 2.04 | ± 0.06 | 2.05 | ± 0.07 | 2.18 | ± 0.07 | 2.17 | ± 0.07 |
| **delta parietal** ^#^ | S2 | 1.42 | ± 0.04 | 1.38 | ± 0.03 | 1.48 | ± 0.02 | 1.48 | ± 0.02 |
|  | SWS | 1.95 | ± 0.06 | 1.98 | ± 0.06 | 2.05 | ± 0.06 | 2.02 | ± 0.04 |
|  |  |  |  |  |  |  |  |  |  |
|  |  | **Physostigmine** | | | | **Placebo** | | | |
|  |  | Odor |  | Vehicle |  | Odor |  | Vehicle |  |
| **SS frontal** | S2 | 0.56 | ± 0.03 | 0.56 | ± 0.04 | 0.55 | ± 0.03 | 0.57 | ± 0.03 |
|  | SWS | 0.60 | ± 0.05 | 0.60 | ± 0.05 | 0.62 | ± 0.05 | 0.68 | ± 0.06 |
| **SS central** | S2 | 0.47 | ± 0.03 | 0.47 | ± 0.03 | 0.49 | ± 0.03 | 0.48 | ± 0.03 |
|  | SWS | 0.46 | ± 0.03 | 0.45 | ± 0.03 | 0.52 | ± 0.05 | 0.52 | ± 0.04 |
| **SS parietal** | S2 | 0.43 | ± 0.03 | 0.43 | ± 0.03 | 0.44 | ± 0.02 | 0.43 | ± 0.02 |
|  | SWS | 0.36 | ± 0.02 | 0.36 | ± 0.02 | 0.41 | ± 0.03 | 0.42 | ± 0.03 |
|  |  |  |  |  |  |  |  |  |  |
|  |  | **Physostigmine** | | | | **Placebo** | | | |
|  |  | Odor |  | Vehicle |  | Odor |  | Vehicle |  |
| **FS frontal** | S2 | 0.47 | ± 0.03 | 0.46 | ± 0.04 | 0.46 | ± 0.05 | 0.47 | ± 0.06 |
|  | SWS | 0.36 | ± 0.03 | 0.35 | ± 0.03 | 0.35 | ± 0.05 | 0.38 | ± 0.05 |
| **FS central** | S2 | 0.49 | ± 0.03 | 0.47 | ± 0.04 | 0.48 | ± 0.04 | 0.48 | ± 0.05 |
|  | SWS | 0.37 | ± 0.02 | 0.35 | ± 0.02 | 0.36 | ± 0.04 | 0.37 | ± 0.04 |
| **FS parietal** | S2 | 0.48 | ± 0.04 | 0.49 | ± 0.04 | 0.48 | ± 0.04 | 0.47 | ± 0.04 |
|  | SWS | 0.35 | ± 0.02 | 0.36 | ± 0.03 | 0.37 | ± 0.04 | 0.37 | ± 0.04 |

Power spectral density estimates during S2 and Slow Wave Sleep (SWS) in log(V²/Hz). Estimates are given for the slow oscillation (SO, 0.1-1 Hz), delta (1-4 Hz), slow spindle (SS, 9-12 Hz), and fast spindle (FS, 12-15 Hz) frequency bands at frontal, central and parietal sites.

* ANOVA main effects for ‘physostigmine/placebo’ (SO frontal: p = .021, central: p = .019, parietal: p = .025; delta central: p = .040). ^#^ Trends for ANOVA main effects for ‘physostigmine/placebo’ (delta frontal: p = .065, parietal: p = .058). † ANOVA interaction for ‘S2/SWS’ x ‘odor/vehicle’ (SO frontal: p = .046).
